# Prognostic Value of Survivin in Patients with Gastric Cancer: A Systematic Review with Meta-Analysis

**DOI:** 10.1371/journal.pone.0071930

**Published:** 2013-08-02

**Authors:** Jin Long Liu, Wei Gao, Qing Min Kang, Xue Jun Zhang, Shu Guang Yang

**Affiliations:** 1 Department of General Surgery 1, the Affiliated Hospital of Chengde Medical College, Chengde City, Hebei Province, China; 2 Department of Gastroenterology, the Chinese People’s Liberation Army 264th Hospital, Taiyuan City, Shanxi Province, China; Sapporo Medical University, Japan

## Abstract

**Objective:**

The prognostic significance of survivin for the survival of patients with gastric cancer remains controversial. Thus, the objective of this study was to conduct a systematic review of the literature evaluating survivin expression in gastric cancer as a prognostic indicator.

**Methods:**

Relevant literature was searched using PubMed, EMBASE, and Chinese biomedicine databases. A meta-analysis of the association between survivin expression and overall survival in patients with gastric cancer was performed. Studies were pooled and summary hazard ratios (HRs) were calculated. Subgroup analyses were also conducted.

**Results:**

Final analysis of 1365 patients from 16 eligible studies was performed. Combined HR suggested that survivin expression had an unfavorable impact on survival of gastric cancer patients (HR=1.39, 95% CI: 1.16-1.68). The unfavorable impact also appeared significant when stratified according to the studies categorized by patients’ ethnicity, detection methods, type of sample, and HR estimate. The combined HR in the English literature showed an inverse effect on survival (HR=1.40, 95% CI: 1.13-1.75), while HR in the non-English literature did not (HR=1.38, 95% CI: 0.93-2.05). When stratified according to the location of survivin expression, combined HR showed that expression in cytoplasm was significantly associated with poor prognosis of gastric cancer patients (HR=1.46, 95% CI: 1.12-1.90). While expression in nucleus was not significantly associated with poor prognosis (HR=1.29, 95% CI: 0.72-2.31), the heterogeneity was highly significant (chi-squared=11.5, I^2^=74%, p=0.009).

**Conclusions:**

This study showed that survivin expression was associated with a poor prognosis in patients with gastric cancer. Cytoplasmic expression of survivin may be regarded as a prognostic factor for gastric cancer patients. In contrast, survivin expression in nucleus did not have a significant impact on patients’ overall survival.

## Introduction

Despite its declining incidence, gastric cancer is still one of the most common malignant tumors worldwide. Although the majority of the patients at an early stage of gastric carcinoma can be cured by surgery, more than half of those at an advanced stage of the disease die of carcinoma recurrence, even after undergoing curative gastrectomy [1]. At present, the only prognostic system routinely employed for the management of gastric cancer is based on the International Union Against Cancer (UICC) tumor-node metastasis (TNM) staging system [2]. Currently, using clinical parameters alone, we cannot accurately predict the clinical outcome of patients after surgery [3]. The outcome is linked not only to the high disease stage, but also to the biologic aggressiveness of the individual disease, which is characterized by high potential for metastasis and resistance to anticancer therapy [4]. The discovery of molecular biological prognostic factors may aid in a more accurate prediction of clinical outcome and may also reveal novel predictive factors and therapeutic targets [5]. Hundreds of studies have evaluated prognostic markers that are associated with clinical outcomes, typically overall or recurrence-free survival in gastric cancer. Of these, survivin, considered a very important prognostic marker, has been widely investigated.

Survivin is also called baculoviral inhibitor of apoptosis repeatcontaining 5 (BIRC5). It is a member of the inhibitor of apoptosis (IAP) family, which is one of the most cancer-specific proteins identified to date, being unregulated in almost all human tumors. Biologically, survivin inhibits apoptosis, enhances proliferation, and promotes angiogenesis [6–8], which is highly expressed in most human tumors and fetal tissue, but is undetectable in most terminally differentiated cells [9]. The molecular basis for cancer specific over expression of survivin is not completely elucidated. Nonetheless, compelling evidence exists that it may originate from amplification of the survivin locus [10], demethylation of the survivin promoter and exons [11], and increased promoter activity [12] mediated by a variety of oncogenic pathways. Because of a larger difference in expression between normal and malignant tissue and its causal role in cancer development, survivin is currently attracting considerable attention as a cancer prognostic indicator.

The expression of survivin has been reported to be a promising prognostic indicator. Most of the reports have associated it with a worse overall survival of various cancers, such as liver, colorectal, breast, lung, and esophageal cancers. Some of these reports have been confirmed by systematic reviews with meta-analysis [13,14]. However, some have associated survivin with a favorable survival of cancer, such as in pancreatic ductal adenocarcinoma; this has also been confirmed by a systematic review [15]. However, evidence regarding the prognostic value of survivin with respect to overall survival in gastric cancer remains controversial. To clarify this question, we performed this systematic review of the literature with meta-analysis.

## Methodology

### Literature search

Studies were identified via an electronic search of PubMed, EMBASE, and Chinese biomedicine databases using the following keywords: gastric cancer, gastric carcinoma, BIRC5, baculoviral inhibitor of apoptosis repeat-containing 5, and survivin. The search ended on December 21, 2012. The references for articles and reviews were manually searched for additional studies. We also hand-searched the journals that published articles most relevant to this review.

### Inclusion and exclusion criteria

The systematic review generated complete databases from published studies dealing with the prognostic value of survivin in patients with gastric cancer. We placed no restrictions on language of publication. To be eligible for inclusion, studies had to meet the following criteria: (1) they measured survivin expression in gastric cancer with methods such as immunohistochemistry (IHC), reverse transcription-polymerase chain reaction (RT-PCR), or fluorescence in situ hybridization (FISH); (2) they compared overall survival between different expressions of survivin in gastric cancer; (3) they contained a hazard ratio (HR) and 95% confidence interval (CI) for overall survival according to survivin status which either were reported or could be computed from the data presented; (4) the prognostic effect was valued by mortality of the patients; (5) when the same author or group reported results obtained from the same patient population in more than one article, the most recent report or the most informative report was included; and (6) the study quality was evaluated >5 stars according to Newcastle-Ottawa quality assessment scale [16].

The exclusion criteria were as follows: (1) letters, reviews, case reports, conference abstracts, editorials, and expert opinion were excluded; (2) articles in which no information on overall survival was given or HR about overall survival from the given information could not be computed were excluded; and (3) articles in which prognostic effect was valued by recurrence of gastric cancer were excluded.

### Data extraction

Two investigators (Liu J. L. and Gao W.) reviewed all of the research that met the inclusion and exclusion criteria. Data were extracted independently by two investigators (Liu J. L. and Gao W.) using a data extraction sheet. Data extracted concerned first author’s name, year of publication, source of patients, language of publication, number of patients, type of sample, assay method, location of expression, and survival data. In addition, controversial problems were resolved in a meeting called by Kang Q. M.

### Assessment of study quality

Study quality was assessed independently by two investigators (Liu J. L. and Gao W.) by means of reading and evaluating according to the Newcastle-Ottawa quality assessment scale. Briefly, the overall star system assesses three main categories on the following: (1) selection of cohort, (2) comparability of cohort, and (3) ascertainment of outcome. A study can be awarded a maximum of one star for each numbered item within the selection and outcome categories. A maximum of two stars can be given for comparability. The total number of stars was accumulated last, with more stars reflecting higher methodological quality. A study can be awarded a maximum of nine stars.

### Statistical analysis

The primary outcome revealed the overall survival in all populations; the outcome was then stratified by ethnicity of patients, assay method, language of publication, type of sample, HR estimate, and location of expression. HR and 95% CI were used to estimate the impact of survivin expression on overall survival. A combined HR>1 implied a worse survival for the group with survivin expression. This pejorative impact of survivin on survival was considered statistically significant if 95% CI for the combined HR did not overlap 1. If a direct report of HR and 95% CI was not available, estimated value was derived indirectly from Kaplan-Meier curves using the methods described by Tierney et al. [17]. Kaplan-Meier curves were read by Engauge Digitizer version 4.1 (http://digitizer.sourceforge.net/), then the survival data read from Kaplan-Meier curves were entered in the spreadsheet appended to Tierney’s paper [17]. This work was performed by two independent persons to reduce inaccuracy in the extracted survival rates. To assess heterogeneity among the studies, we used the Cochran Q and I^2^ statistics. For the Q statistic, a P value <0.10 was considered statistically significant for heterogeneity [18]. Then the random effects model was calculated according to the DerSimonian-Laird method [19]. Otherwise, the fixed-effects model (Mantel-Haenszel method) was used. For I^2^, a value >50% was considered a measure of severe heterogeneity [20], so the conclusion should be made with discretion, or a combination of HRs was given up. All statistical analyses were performed by Review Manager 5.0 (http://www.cochrane.org). A significant two-way P value for comparison was defined as P <0.05.

## Results

### Literature Selection

**Figure 1:**
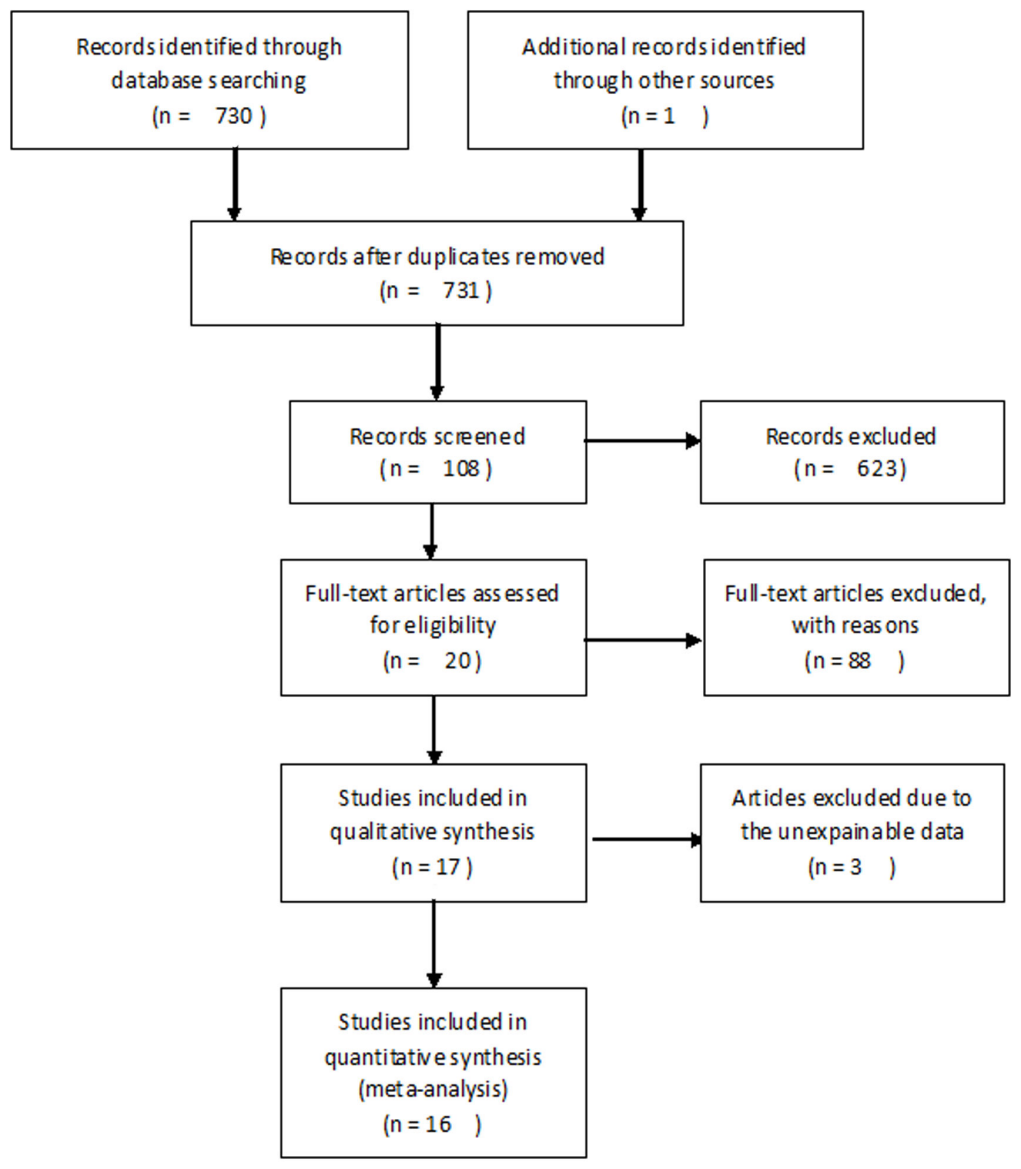
Flow diagram of study selection.

A total of 730 potentially relevant citations were retrieved after the initial search of databases. Additionally, one study was found by hand-search of the journals that published the articles most relevant to this review [21]. The title and abstract of relevant articles were read by the two authors (Liu J. L. and Gao W.) independently. Six hundred twenty-three citations were excluded from analysis after the first screening based on abstracts or titles, leaving 108 available for further full-text review. After carefully reading the full-text articles, 88 studies were excluded; 63 studies were excluded because they were reviews or studies about the correlation with clinicopathological variables, not survival. Another 25 studies were excluded due to insufficient survival data. Last, three studies were excluded due to unexplainable data (data were contradictive in the studies) [22–24]. As a result, 17 eligible studies [21,25–40] were included in the qualitative synthesis, and a final meta-analysis of 1365 patients from 16 evaluable studies was performed (Figure 1).

### Characteristics of the included studies

The basic feature descriptions of the 17 studies are summarized in Table 1. Briefly, study sample sizes ranged from 30 to 157; 14 studies were conducted in Asian populations, while the remaining 3 used European populations [21,25,40]. Fifteen studies investigated survivin by IHC, and two investigated survivin by RT-PCR [25,32]. All of the studies investigated survivin expression using gastric cancer tissues except one study that detected expression using peripheral blood [25]. Eight studies reported survivin as an indicator of poor prognosis, and seven showed no significant impact on overall survival. One study reported survivin as an indicator of good prognosis [40]. In addition, one study reported survivin nuclear staining as a favorable prognosis, while cytoplasmic staining showed no significant impact [33].

**Table 1:** Characteristics and results of included studies.

First Author	Year	NOS	Source	Language	N. of P.	Sample	Method	Location	HR Estimate	HR	95% CIs
Bertazza, L[25]	2009	9	Italy	English	70	peripheral blood	RT-PCR		HR	1.34	1.14-1.53
Chang, J. L[26]	2006	6	Taiwan	English	56	Cancer Tissue	IHC	Nu.	Sur. Curve	1.37	0.77-2.47
Cheng, P[27]	2008	7	China	Chinese	70	Cancer Tissue	IHC	Nu.	Sur. Curve	2.39	1.02-5.56
Deng, J. Y[28]	2010	6	China	English	53	Cancer Tissue	IHC	Cyt.	Sur. Curve	2.4	1.05-5.47
Lee, Gi-Hoon[29]	2006	8	Korea	English	106	Cancer Tissue	IHC	Cyt.	Sur. Curve	1.72	1.01-2.93
Li, Y[30]	2010	8	China	English	65	Cancer Tissue	IHC	NA	Sur. Curve	1.38	0.74-2.57
Meng, J. R[31]	2012	9	China	English	90	Cancer Tissue	IHC	Cyt.	Sur. Curve	1.55	1.00-2.41
Nakamura, M[32]	2004	6	Japan	English	42	Cancer Tissue	RT-PCR		Sur. Curve	1.46	0.64-3.30
Okada, E[33]	2001	8	Japan	English	133	Cancer Tissue	IHC	Nu.	Sur. Curve	0.56	0.32-1.00
						Cancer Tissue	IHC	Cyt	Sur. Curve	0.87	0.46-1.65
Song, K. Y[34]	2009	7	Korea	English	157	Cancer Tissue	IHC	Nu.	HR	1.68	1.06-2.66
Bury, J[21]	2012	7	Poland	English	41	Cancer Tissue	IHC	Both	Sur. Curve	3.74	1.55-9.04
Yu JW[37]	2007	7	China	Chinese	125	Cancer Tissue	IHC	Both	HR	0.786	0.41-1.49
Chen SY[35]	2005	8	China	Chinese	96	Cancer Tissue	IHC	Cyt.	Sur. Curve	1.28	0.71-2.31
Sun YH[36]	2003	8	China	Chinese	131	Cancer Tissue	IHC	Cyt.	HR	1.72	1.02-2.88
Zou DM[39]	2009	8	China	Chinese	80	Cancer Tissue	IHC	Cyt.	HR	0.786	0.41-1.49
Zhang JN[38]	2007	7	China	Chinese	50	Cancer Tissue	IHC	Cyt.	Sur. Curve	2.69	1.11-6.49
Vallbohmer, D[40]	2009	8	Germany	English	30	Cancer Tissue	IHC	Cyt.	HR	0.08	0.02-0.36

NOS, Newcastle-Ottawa quality assessment scale; N.of P, number of patients; 95% CIs, 95% confidence interval; HR, hazard ratio; RT-PCR, reverse transcription polymerase chain reaction; IHC, immunohistochemistry; Nu, nucleus; Cyt., Cytoplasm; Sur. Curve, survival curve; NA, not applicable.

### Methodological quality of the studies

For included studies, two authors independently extracted data and assessed methodological quality using the Newcastle-Ottawa quality assessment scale. The scores are shown in Table 1. Luckily, the studies included in our meta-analysis all have high levels of methodological quality (>5 stars on the Newcastle-Ottawa scale).

### Assessment of heterogeneity

**Figure 2:**
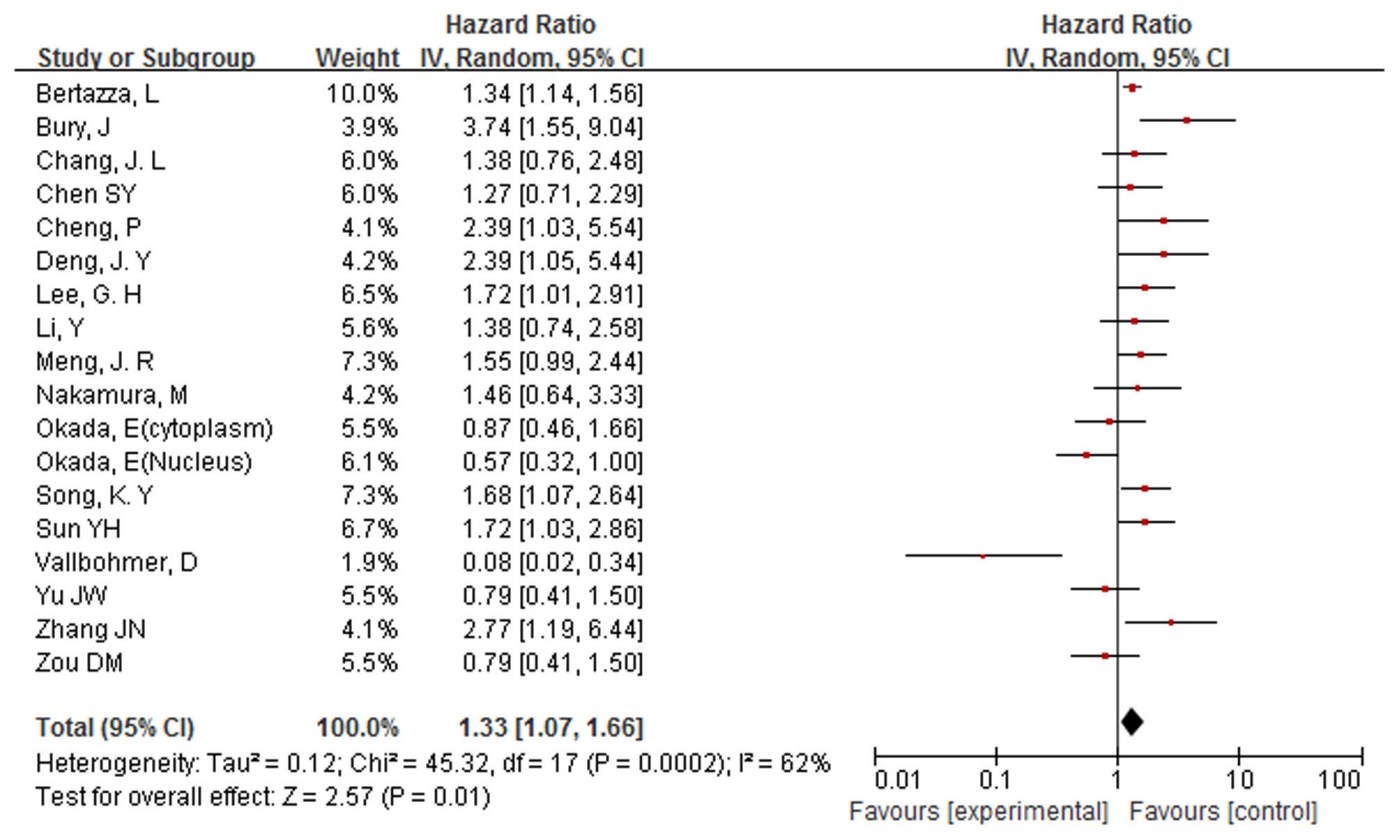
Forest plot of Hazard ratio (HR) for survival of gastric cancer patients. Highly significant heterogeneity can be found before Vallbohmer D’ study was excluded.

Highly significant heterogeneity was detected when all studies were pooled (chi-squared=45.32, I^2^=62%, p=0.0002). Then we easily found the source of heterogeneity from the forest plot (Figure 2): One study examined the association of survivin expression with prognosis in patients with gastric cancer, and all the patients had been treated with aneoadjuvant chemotherapy [40]. In this study, the author reported cytoplasmatic survivin expression as an indicator of good prognosis. We excluded this study from meta-analysis for two reasons: (1) this was the only study in which the patients were treated with chemotherapy before surgical resection and (2) the sample size was small (n=30) and the weight was slight (1.9%), so the impact on the combined HR would be negligible. After excluding it, a final analysis of 1365 patients from 16 evaluable studies was performed. Heterogeneity still existed, but it dropped to the level that random-effects meta-analyses could be conducted without great error (chi-squared=30.67, I^2^=48%, p=0.01).

### Results of meta-analysis

The main results of this meta-analysis are presented in Table 2. Overall, the pooled HR for all evaluable studies on survivin expression in gastric cancer was 1.39 (95% CI: 1.16-1.68). This illustrated that survivin expression was significantly associated with worse overall survival of gastric cancer patients. When grouped according to ethnicity, the combined HR of Asian studies was 1.35 (95% CI: 1.09-1.67). The heterogeneity of the remaining two European studies was highly significant (chi-squared=5.08, I^2^=80%, p=0.02), so the pool of HR was given up to calculate. In the subgroup analysis according to the method of survivin detection used, the combined HR was 1.41 (95% CI: 1.11-1.78) for IHC and 1.34 (95%CI: 1.15-1.56) for RT-PCR. When stratified according to language of publication, the combined HR of English literature showed an inverse effect on survival (HR=1.40, 95% CI: 1.13-1.75), while the non-English literature did not (HR=1.38, 95% CI: 0.93-2.05). When stratified according to type of sample, the combined HR of gastric cancer tissue showed an inverse effect on survival (HR=1.41, 95% CI: 1.13-1.76). When the HRs directly extracted from the five evaluable studies were pooled, the combined HR was 1.29 (95% CI: 1.00-1.66). When the HRs calculated indirectly from Kaplan-Meier-based survival curves were pooled, the combined HR was 1.5 (95% CI: 1.14-1.97). The combined HR from the subgroup indicated that survivin was an independent prognostic marker in gastric cancer.

**Table 2:** Summarized HRs of overall and subgroup analyses for survivin on gastric cancer survival.

	N. of studies	Number of patients	HR(95%CIs)	Heterogeneity test		
				chi-squared	I^2^	P-value
Overall	16	1365	1.39 (1.16-1.69)	30.67	48%	0.01
Ethnicity						
Asian	14	1254	1.35 (1.09-1.67)	25.55	45%	0.03
European	2	111	Given up	5.08	80%	0.02
Methods						
IHC	14	1253	1.41 (1.11-1.78)	30.53	54%	0.01
RT-PCR	2	112	1.34 (1.15-1.56)	0.04	0.00%	0.83
Language						
English	10	813	1.40 (1.13-1.75)	19.85	50%	0.03
Chinese	6	552	1.38 (0.93-2.05)	10.81	54%	0.06
Sample						
Cancer tissue	15	1295	1.41 (1.13-1.76)	30.55	51%	0.01
Peripheral Blood	1	70	1.34 (1.14-1.53)			
HR Estimatie						
HR	5	563	1.29 (1.00-1.66)	7.07	43%	0.13
Sur.Curve	11	802	1.50 (1.14-1.97)	23.13	52%	0.02
Location						
Cytoplasm	8	739	1.46 (1.12-1.90)	10.6	34%	0.16
Nucleus	4	416	1.29 (0.72-2.31)	22.71	52%	0.02

Heterogeneity of European studies was highly sigficant(I^2^ = 80%), so pool of hazard ratio (HR) was given up to calculate.

To further investigate the relationship between survivin subcelluar location and overall survival, eight studies, which reported survivin located in cytoplasm in 739 patients, were enrolled in the meta-analysis [28,29,31,34–36,38,39]. Due to the absence of heterogeneity (chi-squared=10.6, I^2^=34%, p=0.16), a fixed effect model was accepted. The combined HR was 1.46 (95% CI: 1.12-1.90), which demonstrated that positive expression of survivin in cytoplasm was significantly associated with poor prognosis of gastric cancer patients. Four studies reported that survivin expression was located in nucleus in 416 patients [26,27,33,34]. Because of heterogeneity (chi-squared=11.5, I^2^=74%, p=0.009), a random effect model was adopted. The combined HR was 1.29 (95% CI: 0.72-2.31), which illustrated that survivin expression in nucleus was not significantly associated with overall survival of gastric cancer patients. However, the heterogeneity was highly significant (I^2^>50%), so the result should be accepted with discretion.

### Publication bias

**Figure 3:**
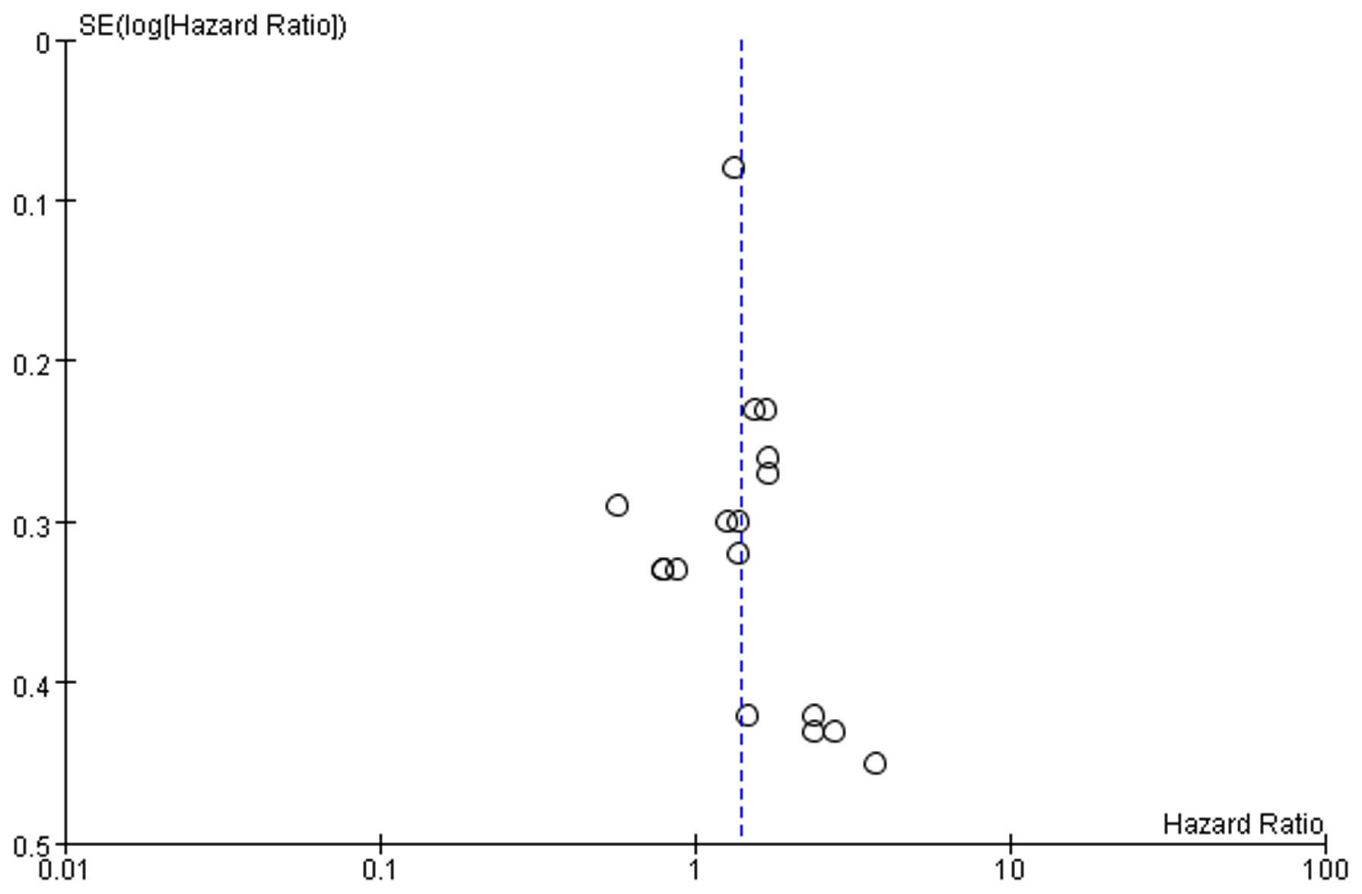
Funnel plots were used to detect publication bias on overall estimate. Studies are distributed symmetrically, and suggest that publication bias is absence in the meta-analysis.

Publication bias may exist when no significant findings remain unpublished, thus artificially inflating the apparent magnitude of an effect. Funnel plots of our study results are shown in Figure 3. In all included studies, no obvious funnel plot asymmetry was found. Thus, no evidence of publication bias was detected.

## Discussion

Survivin as a biomarker of prognosis in malignancies has generated significant interest. However, the conclusions from published research regarding its prognostic value for different cancers are controversial. Survivin expression is an unfavorable prognostic indicator in esophageal and lung cancers [13,14]. In contrast, a favorable outcome associated with nuclear survivin has been reported for pancreatic ductal adenocarcinoma [15]. Many studies have investigated the prognostic value of survivin in gastric cancer, but all the sample sizes have been small. In addition, reports about prognostic significance of survivin in gastric cancer are controversial. No meta-analyses or review protocols have previously been reported on the prognostic value of survivin in gastric cancer. With a larger sample size, we performed a meta-analysis to evaluate the role of survivin in the prognosis of gastric cancer.

In the review, 17 studies were included based on the criteria. Then, one study was excluded from meta-analysis as a source of highly significant heterogeneity [40]. In the excluded study, the patients with gastric cancer were all treated with chemotherapy before surgical resection; it was the only study that reported the expression of survivin in cytoplasm as an indicator of good prognosis. The reasonable explanation may be that high survivin expression causes increased proliferation activity of tumor cells that are more liable to chemotherapeutic cell damage. However, the conclusion should be confirmed in larger trials because the heterogeneity may also be a result of bias from the small sample size.

Ultimately, we enrolled 16 studies concerning expression of survivin in gastric cancer patients’ overall survival for meta-analysis. In all studies, survivin expression was detected by IHC or RT-PCR. The specimens were gastric cancer tissues or peripheral blood. By meta-analysis of the 16 studies, we identified the pool HR which indicated that survivin was a factor in poor prognosis in gastric cancer. We can explain this result by survivin’s ability to inhibit apoptosis, promote proliferation, and enhance angiogenesis. Because of its involvement in these processes, survivin is likely to be causally involved in tumor progression and, consequently, increased levels would be expected to predict a poor prognosis. As a prognosis factor of gastric cancer, survivin may aid in a more accurate prediction of clinical outcome of gastric cancer and may also be a novel therapeutic target.

The subcellular distribution of survivin appears to alter during progression through the cell cycle. For example, survivin was associated with the microtubule organization center during interphase, centrosomes and mitotic spindles at metaphase, but relocated to midbodies in late telophase [41,42]. To investigate the relationship between survivin subcellular location and overall survival, we analyzed eight studies as a subgroup, in which survivin expression was located in cytoplasm; the result showed that survivin expression in cytoplasm was closely associated with poor prognosis of gastric cancer patients. However, when we analyzed another four studies as a subgroup in which survivin expression was found in nucleus, the result showed no significant impact on patients’ overall survival. The different roles of survivin for prognosis in different locations may indicate that in the cell cycle the phase of the tumor cells may contribute to the prognosis of gastric cancer. While the results may relate to varying specificity of the antibodies used in IHC, further work is necessary to establish whether different locations of survivin are associated with different prognoses of cancer.

By this meta-analysis, we enlarged the sample size, so we could get a relactively convincing conclusion. But some limitations in our meta-analysis still existed for unsolvable reasons.

First, we dealt with numerous heterogeneity problems. Heterogeneity might be contributed by the baseline characteristics of the patients, such as age, histological type, differentiation or disease stage, adjuvant treatment they might have received, the duration of follow-up, and adjustments for other cofactors. It is possible that the results of the meta-analysis could have been influenced by the heterogeneity among the 16 studies. Therefore, we attempted to perform a stratified subgroup analysis according to the characteristics of the patients that could be acquired from the studies. However, there were still limitations in this meta-analysis because some of the characteristics could not be acquired from the studies. All these sources of variability could produce additional inconsistencies and cause potential selection bias. Therefore, our results should be substantiated by further prospective studies.

Second, the technique of detecting survivin may lack comparability among the studies. Most studies (87.5%) in the meta-analysis used IHC staining to study expressions of survivin. Although IHC staining is simple and cost-effective to perform, results are highly dependent on a variety of methodological factors, such as storage time, fixation method of paraffin-embedded tissues, different primary antibodies, the revelation protocols, and different levels of positive [43].

Another potential source of bias is related to the method used to extrapolate the HR. If the HR was not reported in a study, it was calculated from the data included in the article or extrapolated from the survival curves. In fact, the method of extrapolating HR from survival curves did seem to be less reliable than when HR was obtained from published statistics because this strategy did not completely eliminate inaccuracy in the extracted survival rates.

Finally, in our study, we did not take into account unpublished articles and abstracts. In addition, of the 731 studies gathered, 25 were not included in the meta-analysis due to a lack of available, or calculated, survival statistics. For these limitations, the pooled HRs calculated in our meta-analysis may be overestimated, and the strength of this study may be weakened.

## Conclusions

Survivin expression was associated with a poor prognosis in patients with gastric cancer in this systematic review with meta-analysis. Cytoplasmic expression of survivin may be regarded as a prognostic factor for gastric cancer patients based on the currently obtained data. In contrast, survivin expression in nucleus does not have a significant impact on patients’ overall survival. Our conclusions should be confirmed by an adequately designed prospective study and the exact role of survivin expression needs to be determined by an appropriate multivariate analysis taking into account the classic well-defined prognostic factors for gastric cancer; in particular, its subcellular location should be carefully considered.

## Supporting Information

Checklist S1 Prisma checklist.
(DOC)
